# A disproportionality analysis on proprotein convertase subtilisin/kexin type 9 inhibitors and hypersensitivity and anaphylaxis

**DOI:** 10.1038/s41598-025-24945-1

**Published:** 2025-11-20

**Authors:** Foteini Dermiki-Gkana, Christopher A. Gravel, Christos Kontogiorgis, Antonios Douros

**Affiliations:** 1 Laboratory of Hygiene and Environmental Protection, Department of Medicine, Democritus University of Thrace, Campus (Dragana), Alexandroupolis, Greece; 2 School of Epidemiology and Public Health, University of Ottawa, Ottawa, ON Canada; 3 Department of Epidemiology, Biostatistics and Occupational Health, McGill University, Montreal, QC Canada; 4 Data Literacy Research Institute, University of Ottawa, Ottawa, ON Canada; 5 Department of Mathematics and Statistics, University of Ottawa, Ottawa, ON Canada; 6 Institute of Clinical Pharmacology and Toxicology, Charité-Universitätsmedizin Berlin, Berlin, Germany

**Keywords:** Cardiovascular diseases, Cardiology, Medical research

## Abstract

Recent case reports linked use of the lipid-lowering class of proprotein convertase subtilisin/kexin type 9 (PCSK9) inhibitors to severe hypersensitivity reactions. Therefore, our pharmacovigilance study assessed the association between reporting of PCSK9 inhibitors and hypersensitivity or anaphylaxis. We analyzed the US Food and Drug Administration Adverse Event Reporting System (FAERS) extracting spontaneous reports from 2015 to 2023. We calculated reporting odds ratios (ROR), proportional reporting ratios, and information components (IC) as measures of disproportionate reporting of hypersensitivity or anaphylaxis with PCSK9 inhibitors overall and with specific compounds (alirocumab, evolocumab) using the entire FAERS as comparator. In sensitivity analyses, we adjusted for demographic characteristics, used statins as active comparator, and applied alternate outcome definitions. Among all reports in FAERS during the study period involving PCSK9 inhibitors, we identified 12,591 cases of hypersensitivity and 17,214 cases of anaphylaxis. Across disproportionality analysis methods, we did not observe an association between the reporting of PCSK9 inhibitors or evolocumab specifically and the outcomes. However, there were associations with alirocumab and hypersensitivity (ROR, 1.14; 95% confidence interval [CI], 1.11–1.18 / IC, 0.19; 95% credible interval [CrI], 0.14–0.24) and with alirocumab and anaphylaxis (ROR, 1.30; 95% CI, 1.26–1.33; IC, 0.37; 95% CrI, 0.33–0.42). Sensitivity analyses corroborated the lack of an association with PCSK9 inhibitors or with evolocumab and were inconsistent regarding alirocumab. Our large pharmacovigilance study showed no signal of disproportionate reporting for hypersensitivity or anaphylaxis with PCSK9 inhibitors overall. The inconsistencies in alirocumab related findings argue against a compound specific signal.

**Supplementary Information:**

The online version contains supplementary material available at 10.1038/s41598-025-24945-1.

## Introduction

Proprotein convertase subtilisin/kexin type 9 (PCSK9) inhibitors include the fully human monoclonal antibodies alirocumab and evolocumab and are used in the management of hypercholesterinemia^1^. PCSK9 inhibitors reduce cholesterol levels and have improved clinical outcomes such as cardiovascular death in randomized controlled trials (RCTs)^2–4^. Overall, PCSK9 inhibitors appear to have a favorable safety profile^1^. However, every third patient who received evolocumab or alirocumab in RCTs developed injection-site reactions^2–4^. Moreover, a meta-analysis of RCTs showed that the risk of injection-site reactions was increased by 68% with alirocumab and by 64% with evolocumab when compared to placebo^5^.

A drug utilization study characterizing the early patterns of use of PCSK9 inhibitors in Denmark reported not only injection-site reactions but also a case of severe hypersensitivity with angioedema^6^. In addition, a recent case report described a hypersensitivity of immediate-type with urticaria after evolocumab treatment with cross-reactivity to alirocumab^7^. This is not surprising; while fully human antibodies such as PCSK9 inhibitors tend to have lower immunogenicity than chimeric or humanized antibodies, hypersensitivity reactions may still occur, although the reasons are not always clear^8^.

Overall, these data allude to moderate-to-severe hypersensitivity as a potential safety issue with PCSK9 inhibitors^9^, leading to concerns and recent labeling changes by regulatory agencies such as the United States Food and Drug Administration (FDA)^10^. However, data on the hypersensitivity potential of PCSK9 inhibitors outside of the restricted setting of RCTs remain scarce and limited. Therefore, more research is needed to better understand this potential association. As a first step to address this important knowledge gap, we conducted a large pharmacovigilance study in a global spontaneous reports database to assess whether the reporting of PCSK9 inhibitors is associated with hypersensitivity or anaphylaxis.

## Methods

### Data source

For this study, we used data from the United States FDA Adverse Event Reporting System (FAERS) database^11^. FAERS is a publicly accessible system where healthcare providers, patients, and pharmaceutical companies submit suspected adverse drug reaction reports. FAERS data are aggregated quarterly and can be extracted in seven different data files that are linkable via a unique identifying number (CASEID), including the following: (i) demographic file with patient information; (ii) drug file with information about the suspected role of the drug in the adverse event (e.g., primary suspect, secondary suspect); (iii) reaction file with clinical information about the adverse event (coded using Medical Dictionary for Regulatory Activities [MedDRA] terminology at the level of ‘preferred terms’); (iv) outcome file with information about the severity of the adverse event and potential complications (e.g., leading to hospitalization or disability, life-threatening, death), (v) report source file (e.g., healthcare professionals, consumers, distributors); (vi) therapy dates file with start and end dates for the reported drugs; and (vii) indications for use file with information on the underlying indications for the reported drugs (coded using MedDRA preferred terms). Given that FAERS data are anonymized, no informed consent or ethics approval were required.

### Study design

We conducted a pharmacovigilance study by extracting and analyzing raw data submitted to FAERS between July 2015 (when the FDA approved alirocumab as the first PCSK9 inhibitor) to June 2023. First, we proceeded to data cleaning according to the FDA guidelines by removing duplicate records via the selection of the most updated entry for each report based on the unique identifying number CASEID and FDA_DT, which is the date of each report or event within the dataset. Then, we identified all reports of hypersensitivity and anaphylaxis linked to PCSK9 inhibitors in the database.

### Exposure definition

We considered the PCSK9 inhibitors alirocumab and evolocumab in our analyses. Inclisiran, which is not an antibody but a small interfering mRNA that inhibits intracellular PCSK9, was not considered given its expected lack of hypersensitivity potential and also its very recent approval. Given the lack of standardized drug names within FAERS, we used both generic names and brand names in our search: “Alirocumab”, “Praluent”, “Praluent Single-Dose Pen”, “Praluent Multi-Dose Pen”, “Εvolocumab”, “Repatha”, “Repatha Pushtronex”, and “Evolocumab Injection”.

### Outcome definition

The primary study outcome was hypersensitivity, which was defined using all preferred terms included in the broad version of the Standardized MedDRA Query (SMQ) “Hypersensitivity” (Supplementary Table 1). Τhe secondary study outcome was anaphylaxis, which was also defined using all preferred terms included in the broad version of the SMQ “Anaphylactic reaction” (Supplementary Table 2). In general, SMQs facilitate the retrieval of MedDRA-coded data comprising pre-determined sets of MedDRA preferred terms that represent signs, symptoms, investigations or diagnoses likely to be relevant to a defined medical condition.

### Statistical analysis

We summarized the characteristics of the reports linking PCSK9 inhibitors to the two outcomes. In our statistical analyses, we utilized commonly used frequentist and Bayesian approaches to assess the potential association between the reporting of PCSK9 inhibitors and hypersensitivity or anaphylaxis^12,13^. To this end, we calculated (i) the proportional reporting ratio (PRR) with 95% confidence intervals (CIs)^14^, where a signal is flagged when the lower bound of the 95% CI exceeds 1, (ii) the reporting odds ratio (ROR) with 95% CIs^15^, where a signal is flagged when the lower bound of the 95% CI exceeds 1, and (iii) the information component (IC) with 95% credible intervals (CrIs)^16^, where a signal is flagged when the lower bound of the 95% CrI exceeds 0. The PRR compares the reporting rates of a specific adverse event in those who reported the drug of interest with the drugs in the reference set. The ROR compares the odds of reporting of an adverse event with the drug of interest to the odds of the same adverse event with the drugs in the reference set. Finally, the IC is the disproportionality measure used in the Bayesian confidence propagation neural network algorithm.

### Additional analyses

We conducted one pre-specified secondary analysis, stratifying by individual PCSK9 inhibitor (alirocumab, evolocumab). Given that the dates of FDA approval for alirocumab and evolocumab were very close, no additional restrictions of the study period were required. We also conducted three pre-specified sensitivity analyses in order to assess the robustness of our findings to specific biases. First, we adjusted our models for age and sex to reduce confounding due to demographic characteristics. Due to the absence of accessible data for imputation in the reports, only complete case reports were included noting that the missingness was likely not at random. Second, given the ongoing uncertainty regarding the most appropriate reference set in pharmacovigilance^17^, we repeated the primary analyses using statins, drugs also used in the management of hypercholesterinemia, as an active comparator. This approach, which was first developed in pharmacoepidemiology, aims to reduce confounding by indication^19^. Third, we repeated the primary analyses using the narrow versions of the SMQs for hypersensitivity and anaphylactic reaction (Supplementary Table 1, Supplementary Table 2), thereby aiming to maximize the specificity of our outcome definitions.

Finally, we performed three post-hoc sensitivity analyses. First, we removed 21 reports that probably referred to the same case. The potentially duplicate reports, that all had different CASEIDs, were identified after manual inspection. Second, we used the algorithmic version of the SMQ for anaphylactic reaction. Third, we repeated all sensitivity analyses separately for alirocumab and for evolocumab. The study was conducted according to The REporting of A Disproportionality Analysis for DrUg Safety Signal Detection Using Individual Case Safety Reports in PharmacoVigilance (READUS-PV) statement (READUS-PV checklist in Supplementary Table 3). We used MedDRA version 28.0. All analyses were performed using R Studio (Vienna, AT).

## Results

After restricting FAERS to the study period 2015–2023 and excluding reports from lawyers (Supplementary Fig. 1), we identified 12,591 reports linking the use of PCSK9 inhibitors to hypersensitivity and 17,214 reports linking the use of PCSK9 inhibitors to anaphylaxis. Evolocumab was the PCSK9 inhibitor most frequently reported in relation to hypersensitivity (*n* = 9,558; 76%) and also in relation to anaphylaxis (*n* = 13,146; 76%). In hypersensitivity reports with PCSK9 inhibitors, mean (standard deviation) age was 67 (10) years, and 61% of patients were female (Table 1). In anaphylaxis reports with PCSK9 inhibitors, mean (standard deviation) age was 67 (10) years, and 60% of patients were female (Table 1). For both study outcomes, all reports classified PCSK9 inhibitors as the primary suspect drug; most reports came from the United States.

**Table 1:** Patient characteristics of cases of hypersensitivity and anaphylaxis with reported use of PCSK9 inhibitors.

Characteristics	Hypersensitivity (*n* = 12,591)	Anaphylaxis (*n* = 17,214)
Age in years, mean (SD)	66.9 (10.0)	67.0 (10.0)
Missing, n (%)	3,925 (31.2)	4,932 (28.7)
Female sex, n (%)	7,454 (63.5)	9,970 (61.5)
Missing, n (%)	844 (6.7)	1,002 (5.8)
**PCSK9 inhibitors, n (%)**		
Alirocumab	3,163 (25.1)	4,195 (24.2)
Evolocumab	9,558 (75.9)	13,146 (75.8)
**Role of PCSK9 inhibitors, n (%)**		
Primary suspect drug	12,591 (100)	17,214 (100)
Secondary suspect drug	0	0
**Outcome, n (%)**		
Hospitalization	715 (5.7)	1,194 (6.9)
Life-threatening	109 (0.9)	162 (0.9)
Death	86 (0.7)	158 (0.9)
Missing n (%)	9,421 (74.8)	12,998 (75.5)
**Reported country, n (%)**		
United States	11,681 (92.8)	16,044 (93.2)
Other	910 (7.2)	1,170 (6.8)

SD, standard deviation; PCSK9, Proprotein convertase subtilisin/kexin type 9.

Table 2 shows that the reporting of PCKS9 inhibitors was not associated with hypersensitivity (PRR, 0.71; 95% CI, 0.70 to 0.74 / ROR, 0.68; 95% CI, 0.67 to 0.69 / IC, -0.49; 95% CrI: -0.52 to -0.46) or anaphylaxis (PRR, 0.83; 95% CI, 0.82 to 0.84 / ROR, 0.80; 95% CI, 0.79 to 0.82 / IC, -0.26; 95% CrI, -0.29 to -0.24) across disproportionality analysis methods. However, the reporting of alirocumab was associated with hypersensitivity (PRR, 1.14; ROR, 1.17; IC, 0.19; all statistically significant) and anaphylaxis (PRR, 1.30; ROR, 1.38; IC, 0.37; all statistically significant). No associations were observed with evolocumab.

**Table 2:** Reporting of PCKS9 inhibitors and hypersensitivity or anaphylaxis (primary and secondary analyses).

	Reports of drug and AE	PRR (95% CI)	ROR (95% CI)	IC (95% CrI)
**Primary analyses**				
Hypersensitivity *				
PCSK9 inhibitors	12,591	0.71 (0.70 to 0.72)	0.68 (0.67 to 0.69)	-0.49 (-0.52 to -0.46)
**Anaphylaxis** *				
PCSK9 inhibitors	17,214	0.83 (0.82 to 0.84)	0.80 (0.79 to 0.82)	-0.26 (-0.29 to -0.24)
**Secondary analyses**				
**Hypersensitivity** *				
Alirocumab	3,163	**1.14 (1.11 to 1.18)**	**1.17 (1.13 to 1.21)**	**0.19 (0.14 to 0.24)**
Evolocumab	9,558	0.64 (0.62 to 0.65)	0.60 (0.59 to 0.61)	-0.65 (-0.68 to -0.62)
**Anaphylaxis** *				
Alirocumab	4,195	**1.30 (1.26 to 1.33)**	**1.38 (1.33 to 1.42)**	**0.37 (0.33 to 0.42)**
Evolocumab	13,146	0.75 (0.74 to 0.76)	0.72 (0.70 to 0.73)	-0.41 (-0.44 to -0.39)

PCSK9, Proprotein convertase subtilisin/kexin type 9; AE, adverse event; PRR, proportional reporting ratio; CI, confidence interval; ROR, reporting odds ratio; IC, information component; CrI, credible interval; SMQ, Standardised Medical Dictionary for Regulatory Activities Query.

* We used the broad versions of SMQs for “Hypersensitivity” and “Anaphylactic Reaction”.

Significant values are in bold.

Table 3 shows the results of the sensitivity analyses for PCSK9 inhibitors overall. The adjustment for demographic characteristics, the use of statins as an active comparator, the use of alternative outcome definitions, and the manual removal of potentially duplicate reports corroborated the results of the primary analysis regarding the lack of an association between the reporting of PCSK9 inhibitors and hypersensitivity or anaphylaxis. Table 4 shows the results of the sensitivity analyses for alirocumab. Some, but not all analyses alluded to an association between the reporting of alirocumab and hypersensitivity. The results of the analyses on anaphylaxis were even less consistent. Table 5 shows the results of the sensitivity analyses on evolocumab, which corroborated the lack of an association between its reporting and the study outcomes.

**Table 3:** Reporting of PCKS9 inhibitors and hypersensitivity or anaphylaxis (sensitivity analyses).

Drug exposure	Reports of drug and AE	PRR (95% CI)	ROR (95% CI)	IC (95% CrI)
**Hypersensitivity**				
Adjusted *	8,608	NA	0.65 (0.52 to 0.80)	NA
Active comparator **	156	0.80 (0.72 to 0.96)	0.80 (0.67 to 0.95)	-0.26 (-0.48 to -0.04)
Stricter outcome definition ***	7,453	0.68 (0.67 to 0.70)	0.66 (0.65 to 0.68)	-0.54 (-0.58 to -0.51)
Duplicate removal ****	12,572	0.71 (0.70 to 0.72)	0.68 (0.66 to 0.69)	-0.49 (-0.52 to -0.47)
**Anaphylaxis**				
Adjusted *	12,203	NA	0.73 (0.60 to 0.88)	NA
Active comparator **	212	1.11 (0.99 to 1.25)	1.15 (0.98 to 1.34)	0.15 (-0.04 to 0.34)
Stricter outcome definition ***	143	0.13 (0.11 to 0.15)	0.13 (0.11 to 0.15)	-2.93 (-3.19 to -2.67)
Duplicate removal ****	17,195	0.83 (0.82 to 0.84)	0.80 (0.79 to 0.82)	-0.27 (-0.29 to -0.24)
Algorithmic definition *****	372	0.25 (0.22 to 0.27)	0.24 (0.22 to 0.27)	-2.01 (-2.17 to -1.85)

PCSK9, Proprotein convertase subtilisin/kexin type 9; AE, adverse event; PRR, proportional reporting ratio; CI, confidence interval; NA, not applicable; ROR, reporting odds ratio; IC, information component; CrI, credible interval; SMQ, Standardised Medical Dictionary for Regulatory Activities Query.

* Adjusted for age and sex; only complete cases included.

** Statins were used as reference group (active comparator) instead of the entire FAERS dataset.

*** Narrow definition of the respective SMQ.

**** After the removal of potentially duplicate reports.

***** Algorithmic definition of the respective SMQ.

**Table 4:** Reporting of alirocumab and hypersensitivity or anaphylaxis (sensitivity analyses).

Drug exposure	Reports of drug and AE	PRR (95% CI)	ROR (95% CI)	IC (95% CrI)
**Hypersensitivity**				
Adjusted *	1,997	NA	1.19 (0.87 to 1.62)	NA
Active comparator **	107	**1.35 (1.15 to 1.58)**	**1.48 (1.19 to 1.86)**	**0.42 (0.17 to 0.68)**
Stricter outcome definition ***	1,776	1.04 (1.00 to 1.09)	1.05 (1.00 to 1.10)	0.06 (-0.01 to 0.13)
Duplicate removal ****	3,144	**1.14 (1.10 to 1.17)**	**1.16 (1.12 to 1.21)**	**0.18 (0.13 to 0.23)**
**Anaphylaxis**				
Adjusted *	2,780	NA	**1.36 (1.02 to 1.81)**	NA
Active comparator **	105	**1.29 (1.10 to 1.52)**	**1.41 (1.12 to 1.76)**	**0.37 (0.11 to 0.62)**
Stricter outcome definition ***	44	0.26 (0.19 to 0.35)	0.26 (0.19 to 0.34)	-1.95 (-2.42 to -1.47)
Duplicate removal ****	4,176	**1.29 (1.26 to 1.33)**	**1.37 (1.32 to 1.42)**	**0.37 (0.32 to 0.41)**
Algorithmic definition *****	99	0.42 (0.34 to 0.51)	0.42 (0.34 to 0.51)	-1.25 (-1.56 to -0.94)

PCSK9, Proprotein convertase subtilisin/kexin type 9; AE, adverse event; PRR, proportional reporting ratio; CI, confidence interval; NA, not applicable; ROR, reporting odds ratio; IC, information component; CrI, credible interval; SMQ, Standardised Medical Dictionary for Regulatory Activities Query.

* Adjusted for age and sex; only complete cases included.

** Statins were used as reference group (active comparator) instead of the entire FAERS dataset.

*** Narrow definition of the respective SMQ.

**** After the removal of potentially duplicate reports.

***** Algorithmic definition of the respective SMQ.

Significant values are in bold.

**Table 5:** Reporting of evolocumab and hypersensitivity or anaphylaxis (sensitivity analyses).

Drug exposure	Reports of drug and AE	PRR (95% CI)	ROR (95% CI)	IC (95% CrI)
**Hypersensitivity**				
Adjusted *	6,648	NA	0.57 (0.46 to 0.71)	NA
Active comparator **	118	0.86 (0.73 to 1.01)	0.83 (0.68 to 1.01)	-0.22 (-0.47 to 0.04)
Stricter outcome definition ***	5,729	0.62 (0.61 to 0.64)	0.60 (0.58 to 0.62)	-0.68 (-0.72 to -0.64)
Duplicate removal ****	9,539	0.64 (0.62 to 0.65)	0.60 (0.59 to 0.61)	-0.65 (-0.68 to -0.62)
**Anaphylaxis**				
Adjusted *	9,466	NA	0.64 (0.52 to 0.79)	NA
Active comparator **	176	**1.26 (1.11 to 1.43)**	**1.35 (1.14 to 1.61)**	**0.33 (0.13 to 0.53)**
Stricter outcome definition ***	102	0.11 (0.09 to 0.13)	0.11 (0.09 to 0.13)	-3.17 (-3.48 to -2.86)
Duplicate removal ****	13,127	0.75 (0.74 to 0.76)	0.71 (0.70 to 0.73)	-0.41 (-0.44 to -0.39)
Algorithmic definition *****	276	0.22 (0.19 to 0.24)	0.21 (0.19 to 0.24)	-2.20 (-2.39 to -2.02)

PCSK9, Proprotein convertase subtilisin/kexin type 9; AE, adverse event; PRR, proportional reporting ratio; CI, confidence interval; NA, not applicable; ROR, reporting odds ratio; IC, information component; CrI, credible interval; SMQ, Standardised Medical Dictionary for Regulatory Activities Query.

* Adjusted for age and sex; only complete cases included.

** Statins were used as reference group (active comparator) instead of the entire FAERS dataset.

*** Narrow definition of the respective SMQ.

**** After the removal of potentially duplicate reports.

***** Algorithmic definition of the respective SMQ.

Significant values are in bold.

## Discussion

In our large pharmacovigilance study, we identified 12,591 reports of hypersensitivity and 17,214 reports of anaphylaxis related to the use of PCKS9 inhibitors. Our primary analysis showed no association between the reporting of PCSK9 inhibitors and the study outcomes, findings that were corroborated in sensitivity analyses. Moreover, the potential signals for alirocumab related hypersensitivity and anaphylaxis were not consistently replicated in sensitivity analyses.

To date, two case reports linking the use of PCSK9 inhibitors to severe hypersensitivity reactions have been published^6,7^. One case referred to PCKS9 inhibitor related angioedema and was reported as part of a drug utilization study; unfortunately, no additional information (e.g., patient characteristics or specific compound) was provided^6^. The other case referred to an immediate-type hypersensitivity following evolocumab treatment with cross reactivity to alirocumab^7^. A 61-year-old male patient diagnosed with hypertension, hyperlipidemia, and coronary artery disease experienced recurrent episodes of urticaria within minutes after each consecutive injection with evolocumab and later with alirocumab. While the rash was initially localized around the injection sites, it progressively worsened after each injection, becoming generalized to the limbs and trunk and only partially responding to antihistamine treatment. IgE-mediated hypersensitivity was confirmed via skin tests^7^.

The pharmacologic mechanism underlying the potential association between the use of PCSK9 inhibitors and the risk of hypersensitivity is not clear. Overall, fully human antibodies such as the PCKS9 inhibitors evolocumab and alirocumab have lower immunogenicity rates when compared to chimeric or humanized antibodies^8,20^. That being said, immunogenicity due to fully human antibodies does exist, since they may retain non-native epitopes^21^. However, this immunogenicity is most often reflected in the development of anti-drug antibodies that can potentially neutralize the therapeutics and affect their efficacy and less in the development of adverse drug effects such as hypersensitivity^20^.

Our findings do not support the generation of a safety signal. First, the effect estimates in some of the alirocumab specific analyses, albeit elevated above the signal detection threshold, were close to the null value, arguing against a strong safety signal. Second, sensitivity analyses addressing potential sources of bias such as outcome misclassification (via the use of alternative outcome definitions) did not corroborate an association between the reporting of alirocumab and hypersensitivity or anaphylaxis.

Our study has strengths. First, this is the first study to assess potential associations between the reporting of PCSK9 inhibitors and hypersensitivity or anaphylaxis, a potential safety issue with this class of relatively novel lipid lowering drugs. Second, the large sample size allowed the precise estimation of measures of disproportionate reporting for both study outcomes related to PCSK9 inhibitors overall and with individual compounds. Third, our statistical analysis plan was comprehensive, since it combined both frequentist and Bayesian approaches for the assessment of potential signals of disproportionate reporting and also multiple sensitivity analyses for the assessment of the impact of different sources of bias.

Our study also has some potential limitations. First, pharmacovigilance studies utilizing spontaneous reporting systems can be affected by reporting biases related both to stimulated reporting but also to underreporting^22^. However, a post-hoc sensitivity analysis where several reports possibly related to the same case were removed yielded consistent findings. Second, confounding by indication or other factors can also affect internal validity. Sensitivity analyses adjusting for demographic characteristics or using statins as an active comparator were overall consistent with the results of the primary analyses. That being said, statins are not ideal comparators for PCSK9 inhibitors given their use at different stages of disease. Third, while SMQ creation is a meticulous process with SMQs being grouped together after extensive review, testing, analysis, and expert discussion, we were not able to identify specific metrics of validity such as sensitivity or specificity for the SMQs used in our study (i.e., “Hypersensitivity”, “Anaphylactic reaction”) in the literature. Finally, the results of our post-hoc sensitivity analysis, in which we manually removed potentially duplicate reports should be interpreted with caution, given that such procedure was not conducted in the reference group.

Overall, our large pharmacovigilance study showed no association between the reporting of PCSK9 inhibitors overall or the reporting of evolocumab specifically and hypersensitivity or anaphylaxis. Moreover, while some of the alirocumab specific analyses alluded to weak safety signals, these signals were not consistent in all of the analyses. While pharmacovigilance studies cannot unequivocally exclude the possibility of drug safety risks, our results should provide some reassurance to prescribing physicians and patients.

## Supplementary Information

Below is the link to the electronic supplementary material.

Supplementary Material 1

## Data Availability

The raw data are publicly available here:https://fis.fda.gov/extensions/FPD-QDE-FAERS/FPD-QDE-FAERS.html.
