# Supplementary material for: A disproportionality analysis on proprotein convertase subtilisin/kexin type 9 inhibitors and hypersensitivity and anaphylaxis
Source: Sci Rep. 2025 Nov 20;15:41137. doi: 10.1038/s41598-025-24945-1 (PMC12635233; doi:10.1038/s41598-025-24945-1)
Supplement: Supplementary file 1 — Supplementary Material 1 [file 41598_2025_24945_MOESM1_ESM.docx]

**Content**

[Supplementary Figure 1. Flowchart 2](#_Toc201245885)

[Supplementary Table 1. PTs for the outcome hypersensitivity 3](#_Toc201245886)

[Supplementary Table 2. PTs for the outcome anaphylactic reaction 10](#_Toc201245887)

[Supplementary Table 3. Checklist for READUS PV 12](#_Toc201245888)

# **Supplementary Figure 1. Flowchart**

# **
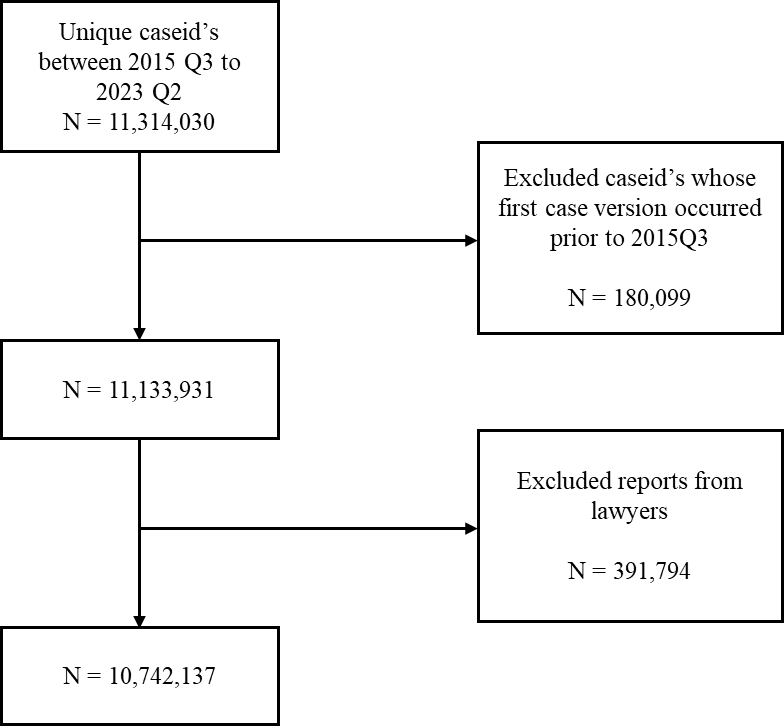
**

# **Supplementary Table 1. PTs for the outcome hypersensitivity**

| **PTs in narrow SMQ definition** | **Additional PTs for broad SMQ definition** |
| --- | --- |
| Acquired C1 inhibitor deficiency | Acute respiratory failure |
| Acute generalised exanthematous pustulosis | Administration site photosensitivity reaction |
| Administration related reaction | Airway remodelling |
| Administration site dermatitis | Allergy to chemicals |
| Administration site eczema | Allergy to fermented products |
| Administration site hypersensitivity | Alpha tumour necrosis factor increased |
| Administration site rash | Alveolitis |
| Administration site recall reaction | Anti-insulin antibody increased |
| Administration site urticaria | Anti-insulin antibody positive |
| Administration site vasculitis | Anti-insulin receptor antibody increased |
| AGEP-DRESS overlap | Anti-insulin receptor antibody positive |
| Allergic bronchitis | Antibody test abnormal |
| Allergic colitis | Antibody test positive |
| Allergic cough | Antibody-dependent enhancement |
| Allergic cystitis | Application site photosensitivity reaction |
| Allergic eosinophilia | Asthma |
| Allergic gastroenteritis | Asthma late onset |
| Allergic hepatitis | Asthma-COPD overlap syndrome |
| Allergic keratitis | Asthmatic crisis |
| Allergic lymphangitis | Auricular swelling |
| Allergic oedema | Blister |
| Allergic otitis externa | Blister rupture |
| Allergic otitis media | Blood immunoglobulin A abnormal |
| Allergic pharyngitis | Blood immunoglobulin A increased |
| Allergic reaction to excipient | Blood immunoglobulin D increased |
| Allergic respiratory disease | Blood immunoglobulin G abnormal |
| Allergic respiratory symptom | Blood immunoglobulin G increased |
| Allergic sinusitis | Blood immunoglobulin M abnormal |
| Allergic stomatitis | Blood immunoglobulin M increased |
| Allergic transfusion reaction | Bronchial hyperreactivity |
| Allergy alert test positive | Bronchial oedema |
| Allergy test positive | Bullous impetigo |
| Allergy to immunoglobulin therapy | Caffeine allergy |
| Allergy to surgical sutures | Capillaritis |
| Allergy to vaccine | Charcot-Leyden crystals |
| Anal eczema | Cheilitis |
| Anaphylactic reaction | Childhood asthma |
| Anaphylactic shock | Choking |
| Anaphylactic transfusion reaction | Choking sensation |
| Anaphylactoid reaction | Complement factor C1 decreased |
| Anaphylactoid shock | Complement factor C1 increased |
| Anaphylaxis treatment | Complement factor C2 decreased |
| Angioedema | Complement factor C2 increased |
| ANCA antibody positive vasculitis | Complement factor C3 decreased |
| Antiallergic therapy | Complement factor C3 increased |
| Antiendomysial antibody positive | Complement factor C4 decreased |
| Application site dermatitis | Complement factor C4 increased |
| Application site eczema | Complement factor decreased |
| Application site hypersensitivity | Complement factor increased |
| Application site rash | Complement fixation abnormal |
| Application site recall reaction | Complement fixation test positive |
| Application site urticaria | Conjunctivitis |
| Application site vasculitis | Corneal exfoliation |
| Arthritis allergic | Cough variant asthma |
| Atopic cough | Cytokine increased |
| Atopy | Cytokine release syndrome |
| Blepharitis allergic | Cytokine storm |
| Blood immunoglobulin E abnormal | Ear swelling |
| Blood immunoglobulin E increased | Enhanced respiratory disease |
| Bone cement allergy | Eosinophil count abnormal |
| Bromoderma Bronchospasm | Eosinophil count increased |
| Bullous haemorrhagic dermatosis | Eosinophil percentage abnormal |
| Catheter site dermatitis | Eosinophil percentage increased |
| Catheter site eczema | Eosinophilia |
| Catheter site hypersensitivity | Eosinophilia myalgia syndrome |
| Catheter site rash | Eosinophilic angiocentric fibrosis |
| Catheter site urticaria | Eosinophilic bronchitis |
| Catheter site vasculitis | Eosinophilic oesophagitis |
| Chronic eosinophilic rhinosinusitis | Eosinophilic pneumonia |
| Chronic hyperplastic eosinophilic sinusitis | Eosinophilic pneumonia acute |
| Circulatory collapse | Eosinophilic pneumonia chronic |
| Circumoral oedema | Erythema |
| Circumoral swelling | Flushing |
| Conjunctival oedema | Foreskin oedema |
| Conjunctivitis allergic | Gastrointestinal oedema |
| Contact stomatitis | Generalised oedema |
| Contrast media allergy | Genital rash |
| Contrast media reaction | Genital swelling |
| Corneal oedema | Haemolytic transfusion reaction |
| Cross sensitivity reaction | HLA marker study positive |
| Cutaneous vasculitis | Human anti-hamster antibody increased |
| Dennie-Morgan fold | Human anti-hamster antibody positive |
| Dermal filler reaction | Immune complex level increased |
| Dermatitis | Immunoglobulins abnormal |
| Dermatitis acneiform | Immunoglobulins increased |
| Dermatitis allergic | Immunology test abnormal |
| Dermatitis atopic | Implant site photosensitivity |
| Dermatitis bullous | Infusion site photosensitivity reaction |
| Dermatitis contact | Injection site panniculitis |
| Dermatitis exfoliative | Injection site photosensitivity reaction |
| Dermatitis exfoliative generalised | Interstitial lung disease |
| Dermatitis herpetiformis | Laryngeal dyspnoea |
| Dermatitis infected | Laryngeal obstruction |
| Dermatitis psoriasiform | Leukotriene increased |
| Device allergy | Lip exfoliation |
| Dialysis membrane reaction | Localised oedema |
| Distributive shock | Macrophage inflammatory protein-1 alpha increased |
| Documented hypersensitivity to administered product | Mechanical urticaria |
| Drug eruption | Medical device site photosensitivity reaction |
| Drug hypersensitivity | Mesenteric panniculitis |
| Drug provocation test | Monocyte chemotactic protein-2 increased |
| DRESS | Mouth ulceration |
| Eczema | Mucocutaneous ulceration |
| Eczema infantile | Mucosa vesicle |
| Eczema nummular | Mucosal erosion |
| Eczema vaccinatum | Mucosal exfoliation |
| Eczema vesicular | Mucosal necrosis |
| Eczema weeping | Mucosal ulceration |
| Encephalitis allergic | Nasal crease |
| Encephalopathy allergic | Necrotising panniculitis |
| Eosinophilic granulomatosis with polyangiitis | Neurodermatitis |
| Epidermal necrosis | Neutralising antibodies positive |
| Epidermolysis | Non-neutralising antibodies positive |
| Epidermolysis bullosa | Noninfective conjunctivitis |
| Epiglottic oedema | Occupational asthma |
| Erythema multiforme | Occupational dermatitis |
| Erythema nodosum | Oedema mucosal |
| Exfoliative rash | Oral mucosal exfoliation |
| Eye allergy | Orbital oedema |
| Eye oedema | Panniculitis |
| Eye swelling | Penile exfoliation |
| Eyelid oedema | Penile oedema |
| Face oedema | Penile swelling |
| Fixed eruption | Perineal rash |
| Generalised bullous fixed drug eruption | Photosensitivity reaction |
| Giant papillary conjunctivitis | Pneumonitis |
| Gingival oedema | Prurigo |
| Gingival swelling | Pruritus |
| Gleich’s syndrome | Pulmonary eosinophilia |
| Haemorrhagic urticaria | Reactive airways dysfunction syndrome |
| Hand dermatitis | Respiratory arrest |
| Henoch-Schonlein purpura | Respiratory distress |
| Henoch-Schonlein purpura nephritis | Respiratory failure |
| Heparin-induced thrombocytopenia | Respiratory tract oedema |
| Hereditary angioedema | Reversible airways obstruction |
| Hereditary angioedema with C1 esterase inhibitor deficiency | Scrotal exfoliation |
| Hypersensitivity | Scrotal swelling |
| Hypersensitivity myocarditis | Seasonal allergy |
| Hypersensitivity pneumonitis | Septal panniculitis |
| Hypersensitivity vasculitis | Skin erosion |
| Idiopathic urticaria | Skin exfoliation |
| Immediate post-injection reaction | Skin oedema |
| Immune thrombocytopenia | Skin swelling |
| Immune tolerance induction | Sneezing |
| Immune-mediated adverse reaction | Stomatitis |
| Implant site dermatitis | Streptokinase antibody increased |
| Implant site hypersensitivity | Stridor |
| Implant site rash | Suffocation feeling |
| Implant site urticaria | Sunscreen sensitivity |
| Incision site dermatitis | Superficial inflammatory dermatosis |
| Incision site rash | Throat tightness |
| Infusion related hypersensitivity reaction | Tongue exfoliation |
| Infusion related reaction | Tracheal obstruction |
| Infusion site dermatitis | Tracheostomy |
| Infusion site eczema | Transplantation associated food allergy |
| Infusion site hypersensitivity | Upper airway obstruction |
| Infusion site rash | Vaccination site photosensitivity reaction |
| Infusion site recall reaction | Vaginal oedema |
| Infusion site urticaria | Visceral oedema |
| Infusion site vasculitis | Vulval oedema |
| Injection related reaction | Vulvovaginal exfoliation |
| Injection site dermatitis | Vulvovaginal swelling |
| Injection site eczema | Wheezing |
| Injection site hypersensitivity |  |
| Injection site rash |  |
| Injection site recall reaction |  |
| Injection site urticaria |  |
| Injection site vasculitis |  |
| Instillation site hypersensitivity |  |
| Instillation site rash |  |
| Instillation site urticaria |  |
| Interstitial granulomatous dermatitis |  |
| Intestinal angioedema |  |
| Iodine allergy |  |
| Kounis syndrome |  |
| Injection related reaction |  |
| Injection site dermatitis |  |
| Injection site eczema |  |
| Injection site hypersensitivity |  |
| Injection site rash |  |
| Injection site recall reaction |  |
| Injection site urticaria |  |
| Injection site vasculitis |  |
| Instillation site hypersensitivity |  |
| Instillation site rash |  |
| Instillation site urticaria |  |
| Interstitial granulomatous dermatitis |  |
| Intestinal angioedema |  |
| Iodine allergy |  |
| Kounis syndrome |  |
| Laryngeal oedema |  |
| Laryngitis allergic |  |
| Laryngospasm |  |
| Laryngotracheal oedema |  |
| Limbal swelling |  |
| Lip oedema |  |
| Lip swelling |  |
| Mast cell activation syndrome |  |
| Mast cell degranulation present |  |
| Medical device site dermatitis |  |
| Medical device site eczema |  |
| Medical device site hypersensitivity |  |
| Medical device site rash |  |
| Medical device site recall reaction |  |
| Medical device site urticaria |  |
| Mouth swelling |  |
| Mucocutaneous rash |  |
| Multiple allergies |  |
| Nephritis allergic |  |
| Nikolsky’s sign |  |
| Nodular rash |  |
| NSAID exacerbated respiratory disease |  |
| Nutritional supplement allergy |  |
| Oculomucocutaneous syndrome |  |
| Oculorespiratory syndrome |  |
| Oedema mouth |  |
| Oral allergy syndrome |  |
| Oropharyngeal blistering |  |
| Oropharyngeal oedema |  |
| Oropharyngeal spasm |  |
| Oropharyngeal swelling |  |
| Palatal oedema |  |
| Palatal swelling |  |
| Palisaded neutrophilic granulomatous dermatitis |  |
| Palpable purpura |  |
| Pathergy reaction |  |
| Penile dermatitis |  |
| Perioral dermatitis |  |
| Periorbital dermatitis |  |
| Periorbital oedema |  |
| Periorbital swelling |  |
| Pharyngeal oedema |  |
| Pharyngeal swelling |  |
| Polymers allergy |  |
| Procedural shock |  |
| Pruritus allergic |  |
| Puncture site rash |  |
| Pustule |  |
| Radioallergosorbent test positive |  |
| Rash |  |
| Rash erythematous |  |
| Rash follicular |  |
| Rash macular |  |
| Rash maculo-papular |  |
| Rash maculovesicular |  |
| Rash morbilliform |  |
| Rash neonatal |  |
| Rash papulosquamous |  |
| Rash pruritic |  |
| Rash pustular |  |
| Rash rubelliform |  |
| Rash scarlatiniform |  |
| Rash vesicular |  |
| Reaction to azo-dyes |  |
| Reaction to colouring |  |
| Reaction to excipient |  |
| Reaction to flavouring |  |
| Reaction to food additive |  |
| Reaction to preservatives |  |
| Reaction to sweetener |  |
| Rhinitis allergic |  |
| Scleral oedema |  |
| Scleritis allergic |  |
| Scrotal dermatitis |  |
| Scrotal oedema |  |
| Serum sickness |  |
| Scleral oedema |  |
| Scleritis allergic |  |
| Scrotal dermatitis |  |
| Scrotal oedema |  |
| Serum sickness |  |
| Serum sickness-like reaction |  |
| Shock |  |
| Shock symptom |  |
| SJS-TEN overlap |  |
| Skin necrosis |  |
| Skin reaction |  |
| Skin test positive |  |
| Solar urticaria |  |
| Solvent sensitivity |  |
| Stevens-Johnson syndrome |  |
| Stoma site hypersensitivity |  |
| Stoma site rash |  |
| Swelling face |  |
| Swelling of eyelid |  |
| Swollen tongue |  |
| Symmetrical drug-related intertriginous and flexural exanthema |  |
| Systemic contact dermatitis |  |
| Tattoo associated skin reaction |  |
| Tongue oedema |  |
| Toxic epidermal necrolysis |  |
| Toxic skin eruption |  |
| Tracheal oedema |  |
| Type I hypersensitivity |  |
| Type II hypersensitivity |  |
| Type III immune complex mediated reaction |  |
| Type IV hypersensitivity reaction |  |
| Urticaria |  |
| Urticaria cholinergic |  |
| Urticaria chronic |  |
| Urticaria contact |  |
| Urticaria papular |  |
| Urticaria physical |  |
| Urticaria pigmentosa |  |
| Urticaria vesiculosa |  |
| Urticarial dermatitis |  |
| Urticarial vasculitis |  |
| Vaccination site dermatitis |  |
| Vaccination site eczema |  |
| Vaccination site exfoliation |  |
| Vaccination site hypersensitivity |  |
| Vaccination site rash |  |
| Vaccination site recall reaction |  |
| Vaccination site urticaria |  |
| Vaccination site vasculitis |  |
| Vaccination site vesicles |  |
| Vaginal ulceration |  |
| Vancomycin infusion reaction |  |
| Vascular access site dermatitis |  |
| Vascular access site eczema |  |
| Vasculitic rash |  |
| Vernal keratoconjunctivitis |  |
| Vessel puncture site rash |  |
| Vessel puncture site vesicles |  |
| Vulval eczema |  |
| Vulval ulceration |  |
| Vulvovaginal rash |  |
| Vulvovaginal ulceration |  |

Abbreviations: PT, preferred term; SMQ, Standardized MedDRA Query; MedDRA, Medical Dictionary for Regulatory Activities; AGEP-DRESS, acute generalized exanthematous pustulosis - drug reaction with eosinophilia and systemic symptoms; COPD chronic obstructive pulmonary disease; ANCA, anti-neutrophil cytoplasmic antibody; HLA, human leukocyte antigen; NSAID, non-steroidal anti-inflammatory drug; SJS-TEN, Stevens–Johnson syndrome - toxic epidermal necrolysis.

# **Supplementary Table 2. PTs for the outcome anaphylactic reaction**

| **PTs in narrow SMQ definition** | **Additional PTs for broad SMQ definition** |
| --- | --- |
| Anaphylactic reaction | Acute respiratory failure |
| Anaphylactic shock | Asthma |
| Anaphylactic transfusion reaction | Bronchial oedema |
| Anaphylactoid reaction | Bronchospasm |
| Anaphylactoid shock | Cardio-respiratory distress |
| Circulatory collapse | Chest discomfort |
| Dialysis membrane reaction | Chocking |
| Kounis syndrome | Chocking sensation |
| Procedural shock | Circumoral oedema |
| Shock | Cough |
| Shock symptom | Cough variant asthma |
| Type I hypersensitivity | Cyanosis |
|  | Dyspnoea |
|  | Enhanced respiratory disease |
|  | Hyperventilation |
|  | Irregular breathing |
|  | Laryngeal dyspnoea |
|  | Laryngeal oedema |
|  | Laryngospasm |
|  | Laryngotracheal oedema |
|  | Mouth swelling |
|  | Nasal obstruction |
|  | Oedema mouth |
|  | Oropharyngeal oedema |
|  | Oropharyngeal spasm |
|  | Oropharyngeal swelling |
|  | Pharyngeal oedema |
|  | Pharyngeal swelling |
|  | Respiratory arrest |
|  | Respiratory distress |
|  | Respiratory dyskinesia |
|  | Respiratory failure |
|  | Reversible airways obstruction |
|  | Sensation of foreign body |
|  | Sneezing |
|  | Stridor |
|  | Swollen tongue |
|  | Tachypnoea |
|  | Throat tightness |
|  | Tongue oedema |
|  | Tracheal obstruction |
|  | Tracheal oedema |
|  | Upper airway obstruction |
|  | Wheezing |
|  | Acquired C1 inhibitor deficiency |
|  | Allergic oedema |
|  | Angioedema |
|  | Circumoral swelling |
|  | Erythema |
|  | Eye oedema |
|  | Eye pruritus |
|  | Eye swelling |
|  | Eyelid oedema |
|  | Face oedema |
|  | Fixed eruption |
|  | Flushing |
|  | Hereditary angioedema with C1 esterase inhibitor deficiency |
|  | Injection site urticaria |
|  | Lip oedema |
|  | Lip swelling |
|  | Nodular rash |
|  | Ocular hyperaemia |
|  | Oedema |
|  | Oedema blister |
|  | Periorbital oedema |
|  | Periorbital swelling |
|  | Pruritus |
|  | Pruritus allergic |
|  | Rash |
|  | Rash erythematous |
|  | Rash pruritic |
|  | Skin swelling |
|  | Swelling |
|  | Swelling of eyelid |
|  | Urticaria |
|  | Urticaria popular |
|  | Blood pressure decreased |
|  | Blood pressure diastolic decreased |
|  | Blood pressure systolic decreased |
|  | Cardiac arrest |
|  | Cardio-respiratory arrest |
|  | Cardiovascular insufficiency |
|  | Diastolic hypotension |
|  | Hypotension |
|  | Hypotensive crisis |
|  | Post procedural hypotension |

Abbreviations: PT, preferred term; SMQ, Standardized MedDRA Query; MedDRA, Medical Dictionary for Regulatory Activities.

# **Supplementary Table 3. Checklist for READUS PV**

| **Section and topic** | **Item #** | **Checklist item** | **Location where item is reported** |
| --- | --- | --- | --- |
| **Title** |  |  |  |
|  | *1a* | *If disproportionality analyses are a prominent component of the published study, the study should be identified as a “disproportionality analysis”. The type of data and name of the database(s) should be specified.* | *1* |
|  | *1b* | *Report the name of adverse event(s) and/or drug(s) under study, when applicable.* | *1* |
| **Introduction** |  |  |  |
| Background | *2a* | *Describe the drug(s) and its utilization, the nature of the adverse event(s) under study and its frequency, and the existing knowledge on the drug-event combination.* | *4* |
|  | *2b* | *Specify the rationale for performing the analysis, e.g., as part of routine pharmacovigilance, to investigate an overall safety profile, or to assess a pre-specified hypothesis.* | *4* |
|  | *2c* | *Explain why ICSR databases and disproportionality analysis are suitable to fill the knowledge gap.* | *4* |
| Objectives | *3* | *State specific objectives, identifying the adverse event(s), the drug(s), and the reference group, including any pre-specified hypothesis, if applicable.* | *4* |
| **Methods** |  |  |  |
| Study design | *4a* | *Identify the study (i.e., “disproportionality analysis”) and the type of data used (e.g., “individual case safety reports”).* | *5* |
|  | *4b* | *Provide an outline of the entire study design, including primary and sensitivity analyses performed, and other designs such as case-by-case analysis or literature review.* | *5* |
| Data description, access, and pre-processing | *5a* | *Specify the name of the database(s), the database(s) custodian, and the coverage. Specify the type/number of drugs included within the database and the thesaurus, taxonomies, or ontologies used for coding drugs and events.* | *5-7* |
|  | *5b* | *Specify the extraction dates and describe and justify all choices used for data pre-processing, including any data transformation or exclusion, if appropriate.* | *5-7* |
| Variables definition | *6a* | *Describe the study population, including any restriction.* | *5-7* |
|  | *6b* | *Describe the nature and the meaning of key variables assessed in the work.* | *5-7* |
|  | *6c* | *Specify and justify any grouping of drugs or events. For drugs, specify and justify whether active ingredients/trade names/salts were considered and/or the selected role.* | *5-7* |
|  | *6d* | *Describe any additional data source used, the type of data, and how they interact with ICSRs.* | *NA* |
| Statistical methods | *7a* | *Present any descriptive analysis performed, specifying variables investigated, statistical tests, and significance thresholds.* | *7* |
|  | *7b* | *Describe the measure(s) selected for the disproportionality analysis including any threshold used to identify signals of disproportionate reporting. Explain the reason for this choice if applicable.* | *7* |
|  | *7c* | *Clearly describe any sensitivity analysis and any tool to control confounding, including any restriction, subgroup, stratification, adjustment, or interaction.* | *7-8* |
|  | *7d* | *Specify the variables and methods used for the case-by-case analysis, including any algorithm or criteria used to assess causality, if performed.* | *NA* |
|  | *7e* | *Specify any statistical methods used for other data sources.* | *NA* |
| **Results** |  |  |  |
| Participants | *8a* | *Specify the number of individual case safety reports included at each stage, including reasons for exclusion.* | *9, Appendix* |
|  | *8b* | *Provide key demographic and clinical characteristics of cases, if possible comparing cases with any appropriate reference group.* | *9, Table 1* |
| Disproportionality analysis | *9* | *Present all results including confidence intervals. Present also results of sensitivity analyses, if performed.* | *9, Tables 2-5* |
| Case-by-case analysis | *10* | *Present the case-by-case analysis of key variables. Present the causality assessment, if applicable.* | *NA* |
| **Discussion** |  |  |  |
| Key results | *11* | *Discuss key results with reference to study objectives and contextualize them within the current literature and other consulted sources. Clearly discriminate between expected reactions and emerging safety signals.* | *11-13* |
| External validity | *12a* | *Discuss the external validity of the results to the general population.* | *11-13* |
|  | *12b* | *Discuss the potential relevance of results in clinical practice* | *11-13* |
|  | *12c* | *Propose further study designs if applicable* | *11-13* |
| Limitations | *13* | *Present general limitations, making clear that disproportionality analysis alone cannot prove causation or measure incidence, and specific limitations, including confounding and reporting bias and efforts to mitigate them.* | *12-13* |
| **Declarations** |  |  |  |
|  | *14a* | *Provide the source of funding/sponsorship and the role of the funders/sponsors for the present study and for any original study on which the present article is based.* | *14* |
|  | *14b* | *Clearly identify potential commercial and intellectual conflicts of interest (e.g., link to any drug/event investigated, whether financial, legal action, or software used).* | *14* |
|  | *14c* | *Declare any institutional approval needed or granted in the investigation.* | *14* |
|  | *14d* | *Include a statement on data availability, code availability (including the version of the statistical software used), and protocol registration.* | *14* |
